# In vitro characterization of a chitosan skin regenerating template as a scaffold for cells cultivation

**DOI:** 10.1186/2193-1801-2-79

**Published:** 2013-03-05

**Authors:** Abu Bakar Mohd Hilmi, Ahmad Sukari Halim, Asma Hassan, Chin Keong Lim, Kartini Noorsal, Ismail Zainol

**Affiliations:** 1 Reconstructive Sciences Unit, Universiti Sains Malaysia, Kelantan, Malaysia; 2 Department of Anatomy, Universiti Sains Malaysia, Kelantan, Malaysia; 3 Universiti Teknologi MARA, Selangor, Malaysia; 4 Standard and Industrial Research Institute of Malaysia (SIRIM), Kedah, Malaysia; 5 Universiti Pendidikan Sultan Idris, Perak, Malaysia

**Keywords:** Chitosan SRT, Interconnected pores, Human dermal fibroblasts, Three dimensional

## Abstract

Chitosan is a marine-derived product that has been widely used in clinical applications, especially in skin reconstruction. The mammalian scaffolds derived from bovine and porcine material have many limitations, for example, prion transmission and religious concerns. Therefore, we created a chitosan skin regenerating template (SRT) and investigated the behavior of fibroblast cell-scaffold constructs. Primary human dermal fibroblasts (HDF) were isolated and then characterized using vimentin and versican. HDF were seeded into chitosan SRT at a density of 3×10^6^ cells/cm^2^ for fourteen days. Histological analysis and live cells imaging revealed that the cell-chitosan constructs within interconnected porous chitosan showed significant interaction between the cells as well as between the cells and the chitosan. Scanning electron microscopy (SEM) analysis revealed cells spreading and covering the pores. As the pore sizes of the chitosan SRT range between 40–140 μm, an average porosity is about 93 ± 12.57% and water uptake ratio of chitosan SRT is 536.02 ± 14.29%, it is a supportive template for fibroblast attachment and has potential in applications as a dermal substitute.

## Background

Chitosan is a biopolymer (β-1,4-D-glucosamine) derivative of chitin (poly-N-acetylglucosamine) that has become an excellent biomedical resource in clinical medicine, especially in dermatology and plastic reconstructive surgery. Chitosan biomaterials are normally used to cover full-thickness skin defects resulting from traumas (Halim et al. 1998; Pusateri et al. 2003), burns (Jin et al. 2007; Guo et al. 2011) and skin ulcers (Anandan et al. 2004; Park et al. 2009). The recent use of chitosan in regenerative medicine, particularly in skin tissue engineering, has been beneficial for the treatment of full-thickness skin defects (Tchemtchoua et al. 2011).

Chitosan was reported to improve homeostasis (Kozen et al. 2008) and promote granulation by enhancing the function of inflammatory cells such as leukocytes, fibroblasts and macrophages (Ueno et al. 2001). Furthermore, chitosan has antimicrobial effects against bacteria, fungi and viruses (Rabea et al. 2003). In addition to the skin, chitosan scaffolds also stimulate the regeneration of spinal cord (Li et al. 2009), cornea (Auxenfans et al. 2009) and liver (Wang et al. 2008) tissues in their respective three-dimensional (3-D) environments.

As a marine product, chitosan has many advantages compared with other collagen biomaterials that are prepared from bovine or porcine sources. These mammalian products have resulted in the transmission of prions to humans. In contrast, marine-derived products are safe for human use due to the species barrier (Prusiner 1998).

Fibroblasts are mesenchymal cells that provide tissue maintenance and support by secreting extracellular matrix (ECM) in connective tissue. In the full-thickness skin model, cultured allogenic fibroblasts in 3-D scaffolds induce inflammation and scar formation, which are important events in wound healing (Lamme et al. 2002). This model has been reported to improve the regeneration of dermis (Lamme et al. 2000) and epidermis (El-Ghalbzouri et al. 2002). Van den Bogaerd et al. (2002) demonstrated that dermis is the best source for skin tissue engineering applications, based on fibroblast yields and the reduced contraction of the wound.

Recently, a study conducted by Lim et al. (2010) showed that chitosan porous skin regenerating templates (SRT) are biocompatible, as determined using primary human keratinocytes with the direct-contact method. They found that chitosan porous SRT did not induce additional inflammatory responses or DNA damage. Their data highlighted the potential use of chitosan porous SRT in skin tissue engineering. However, its potential as a scaffold to support cell attachment and proliferation is still unexplored.

Therefore, this study investigated the ability of a 3-D structure of chitosan porous SRT to support human fibroblast spreading and viability. Chitosan SRT supports fibroblast attachment and has potential for use in skin tissue engineering applications especially in the *de novo* fabrication of dermal substitutes.

## Results and discussion

### Isolation and characterization of HDF

**Figure 1:**
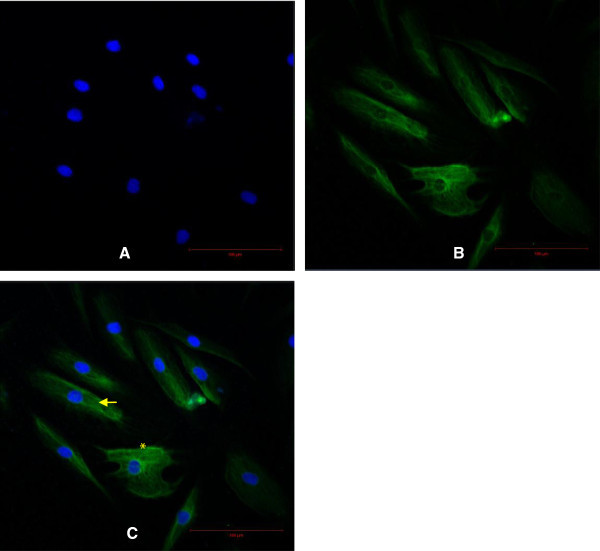
**Characterization of the HDF using versican.** Scale bar 100 μm. Nuclei were stained blue with DAPI (**A**). Cell membrane was stained green with FITC (**B**). The merged image of HDF in polygonal (asterisk) and elongated spindle-shape (arrow) (**C**).

**Figure 2:**
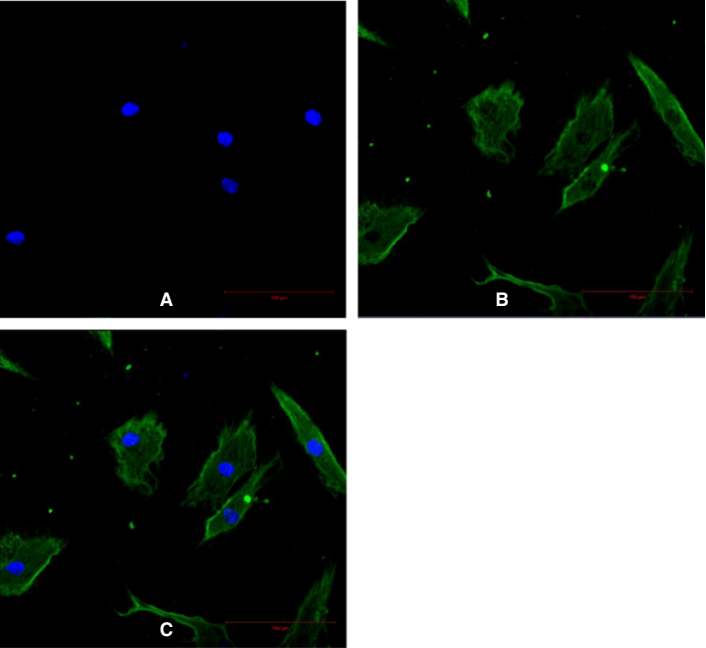
**Characterization of the HDF using vimentin.** Scale bar 100 μm. Nuclei were stained blue with DAPI (**A**). Cell membrane was stained green with FITC (**B**). The merged Image (**C**).

On day 2 after their isolation, primary HDF were observed to be attached to the culture flask and the medium was changed to remove the floating cells and debris. The HDF were spindle-shaped and had polygonal morphology and were confluent on days 10 to 12. Characterization of the HDF was performed by examining versican (Figure 1) a type-lll intermediate filament (IF) protein and vimentin (Figure 2) a chondroitin sulfate proteoglycan. In both assays, the nuclei were stained blue with DAPI and the cell membrane was stained green with fluorescein isothiocyanate (FITC). Vimentin is a mesenchymal marker for which epithelial and endothelial cells are negative (Chang et al. 2002). Vimentin constitute a major portion of the cytoskeleton, has an important role in supporting organelle organization (Katsumoto et al. 1990) and contributes to the plasma membrane fusion machinery in fibroblast (Faigle et al. 2000). As an IF (10 nm filament), vimentin is involved in the formation of membrane proteins such as cystic fibrosis transmembrane conductance regulator (Johnston et al. 1998). Versican is another important marker found abundantly in dermal fibroblasts especially as versican isoform V1 (Hattori et al. 2011). Apart from influencing the dermal fibroblast phenotype, versican has an important role in activating cells adhesion, preventing apoptosis (Wu et al. 2002) and extracellular matrix assembly (Thomas NW, 2002). The characterization of these two markers is important for the future engineering of skin tissue. Dermal fibroblast vimentin^+^ for instance, is an indicator of the multipotency of adult stem cells (Chen et al. 2007) and fibroblast versican^+^ mediates the mesenchymal-epithelial transition (Sheng et al. 2006). Both these characteristics are essential for 3-D tissue regeneration.

### Characterization of chitosan SRT, cell attachment and proliferation in vitro

**Figure 3:**
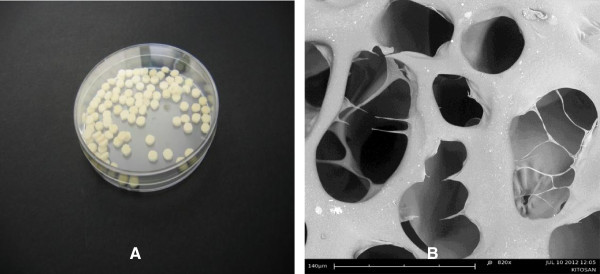
**The macroscopic view of chitosan SRT of 5 mm diameter and 2 mm thickness (A).** Chitosan SRT with interconnected pores. Scale bar 140 μm (**B**).

The macroscopic view of sponge chitosan SRT is shown in Figure 3A. Chitosan SRT is a graded porous scaffold with pore sizes ranging between 40 ± 15.11 μm to 140 ± 18.21 μm in diameter (Figure 3B) and with other mechanical properties described in Table 1. The viability analysis of HDF in 3-D cultures was described in Table 2. The HDF maintained its viability until 14 days of cultures in the chitosan scaffold. Scaffolds with interconnected macroscopic-microscopic pore structures allow the acceleration of tissue regeneration (Peter XM, 2008). Scaffolds with macroscopic pores that are, at least 100 μm in diameter play a role in enhancing ingrowths of cells and blood vessels (Chen and Ma, 2004) while microscopic porosity leads to high cell attachment, proliferation and cellular responses (Ma and Choi, 2001). To allow 3-D tissue regeneration, scaffolds should perform a few critical functions. Firstly, they should provide the cells with a proper surface for attachment and proliferation. Secondly, they should have interconnected pores to allow uniform cells spreading. Lastly, they should provide a 3-D template for specific tissue reconstruction (Zeltinger et al. 2001). Using HDF, the chitosan SRT has performed these functions successfully.

**Table 1:** **Tabulated data for the characterization of chitosan SRT (mean ± SEM, n = 6)**

Properties	Units
Tensile strength	5.02 ± 0.73 N/m^2^
Average pore diameter	
Small pore	39.41 ± 15.11 μm
Large pore	143.55 ± 18.21 μm
Average porosity	93.21 ± 12.57%
Moister flux	3.97 ± 0.34 mg/cm^2^ hr
Water uptake ratio	536.02 ± 14.29%
In vitro degradation	35 ± 5 days

**Table 2:** **OD**_**570**_**value of fibroblasts into chitosan (mean ± SEM, n = 4)**

2 days	4 days	6 days	8 days	10 days	12 days	14 days
0.43±0.04	0.36±0.04	0.50±0.02	0.41±0.02	0.40±0.01	0.35±0.05	0.44±0.02

As a graded porous scaffold, chitosan SRT mimics the natural porous structure more closely than a uniformly porous scaffold. Uniformly porous scaffolds have many limitations. They only allow one cell type to grow depending on the pore size, whereas graded porous scaffold regenerated multiple types of tissue simultaneously (Oh et al. 2007). Yannas et al. (1989) found that the optimum pore size for skin regeneration templates ranged between 20 – 125 μm. Hence, chitosan SRT fulfills this criterion and is suitable as a template for skin tissue engineering.

The ability of chitosan to adsorb nutrients involved in wound healing and exudation of the wound bed is important factors in skin regeneration. Therefore, the determination of water adsorption of chitosan is important to enhance the biological activity of a dermal equivalent. The water uptake ratio of chitosan SRT (536.02 ± 14.29%) was higher than reported previously by Shi et al. (2006). It can attribute to hydrophilicity and the maintenance of 3-D structure. Scaffold with high water uptake ratio is suitable both in full thickness wound that contains excess exudates and chronic inflammation that leading to impair wound healing. To enhance the dermal regeneration and wound closure, it is important to maintain a moist of wound bed. They can be achieved if scaffold has suitable water vapor permeability (WVP). The WVP of chitosan SRT (3.97 ± 0.34 mg/cm^2^ hr) was higher than suggested by Lamke et al. (1977). It happened because of chitosan SRT adsorbed more water. Therefore, more water was vaporized. The WVP depended on scaffold thickness and the ratio of scaffold area to water surface area (Hu et al. 2001). The WVP must be suitable for wound dressing. If the WVP is too high, the wound bed will dry and will increase the metabolism activity. Contrary, if the WVP is too low, the accumulation of exudates will trigger the onset of bacterial growth. The tensile strength of chitosan SRT was determined as 5.02 ± 0.73 MPa. Jansen and Rottier (1958) reported that the tensile strength for male skin ranged between 3 – 14 MPa. Meanwhile, for female skin, the ranged was 4 – 13 MPa. Therefore, chitosan SRT is suitable for both male and female skin regeneration.

**Figure 4:**
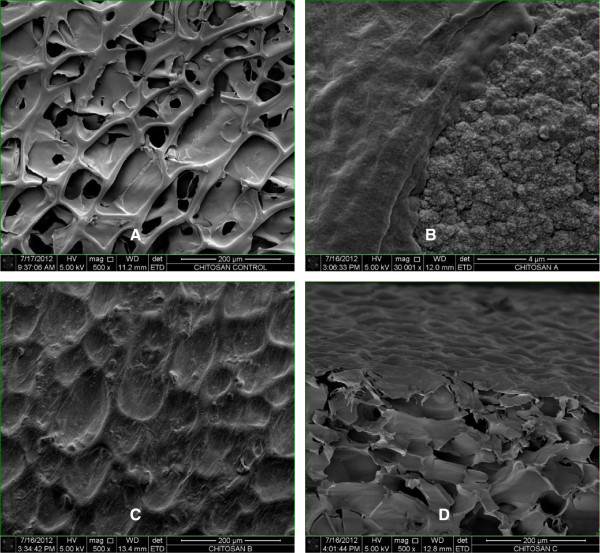
**SEM micrograph of a 3-D culture of HDF into chitosan on day 14.** Chitosan scaffold without cells. Scale bar 200 μm (**A**). Fibroblast proliferation into the chitosan. Scale bar 4 μm (**B**). Fibroblasts covering the pores. Scale bar 200 μm (**C**). Cross section. Scale bar 200 μm (**D**).

**Figure 5:**
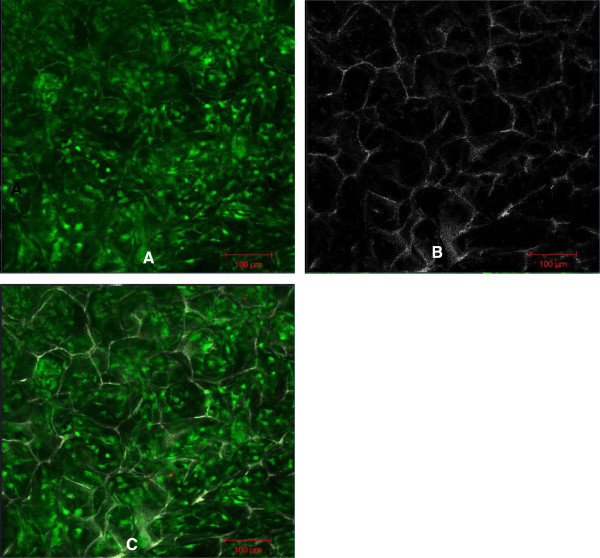
**Confocal micrograph of a 3-D culture of HDF into a chitosan on day 14.** Scale bar 100 μm. Live cell imaging of HDF (**A**). The unstained architecture of the chitosan (**B**). 3-D cultured cells into a chitosan scaffold (**C**).

**Figure 6:**
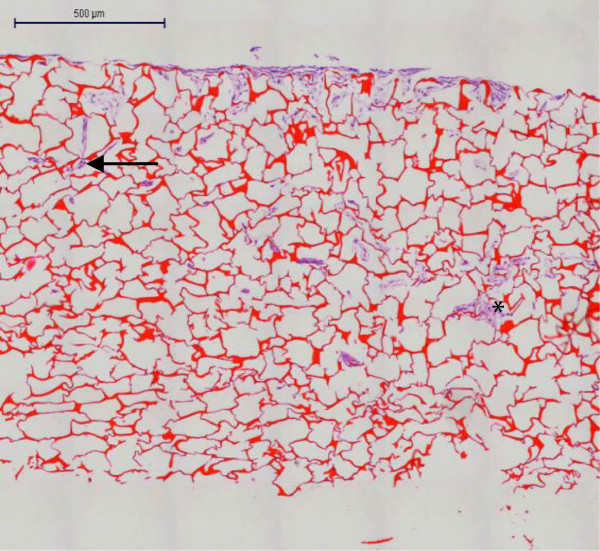
**Histological analysis of HDF in 3-D culture.** HDF integrated to each other (purple, asterisk) and into a chitosan scaffold (purple-red, arrow) on day 14. Scale bar 500 μm.

SEM imaging showed HDF attached to the chitosan and covering most of the pores (Figure 4). Analysis at higher magnification revealed high cell adhesion and layers formation (Figure 4B). Confocal microscopy of the cell-chitosan construct revealed that the live cells were stained with green fluorescence in 3-D cultures (Figure 5). Histological analysis showed that the HDF migrated over and penetrated the chitosan (Figure 6). The interconnected pores facilitate nutrient flux, cell migration and metabolic waste exchange. Cell migration is a primary factor modulating cell behavior and phenotype (Frederick 2003; Even-Ram and Yamada 2005).

## Conclusions

In conclusion, we demonstrated that a chitosan SRT supports HDF attachment. The presence of interconnected pores of both macroscopic and microscopic size in the chitosan is considered as a factor in regulating the survival and attachment of the cell-chitosan construct. Furthermore, the physical and mechanical properties of chitosan SRT are useful for the cultivation of HDF for dermal regeneration. To the best of our knowledge, this is the first microscopic study to analyze the integration of HDF and chitosan SRT.

## Methods

This research was approved by the Research Ethics Committee (Human) of Universiti Sains Malaysia (approval code: USMKK/PPP/JEPeM [212.3(15)]).

### Isolation and characterization of HDF

Skin samples were obtained from ten consenting patients who underwent plastic surgery (according to a protocol approved by an ethics committee and in accordance with the Declaration of Helsinki). To avoid contamination and to maintain cellular integrity, the skin was dissected within 24 hours. It was cut into small pieces, each approximately 0.3 cm^2^ to 0.5 cm^2^, in a Petri dish (Nunc, Denmark) containing phosphate-buffered saline (PBS) (Gibco, USA). PBS was used for washing off cell debris, including fat and coagulated blood. To separate the epidermis and dermis, the skin pieces were incubated in William’s E Medium (Sigma, Germany) supplemented with 0.1% (w/v) reconstituted dispase (Invitrogen, Japan) and 1% (v/v) antimycotic (Invitrogen, USA) overnight at 4°C or for two to three hours at 37°C. After the epidermis was discarded, the dermis was incubated in William’s E Medium (Sigma, Germany) containing 0.1% (w/v) collagenase type I (Gibco, USA) overnight in a 37°C incubator-roller (Binder, Germany). After vigorous resuspension, the cell suspensions were filtered using 70-μm strainers (BD Falcon, USA) to separate the hair shafts and agglutinated cells. The William’s E Medium was dispensed onto the strainer to increase the yield of cells. Human dermal fibroblasts (HDF) were collected by centrifugation (Hettichzentrifugen, Germany) at 420 × *g* for 5 minutes and cultured in 6-well plates (Nunc, Denmark) at a density of 1×10^5^ cells per well in Dulbecco’s Modified Eagle Medium (DMEM) (Invitrogen, USA) supplemented with 1% (v/v) antimycotic and 10% (v/v) fetal bovine serum (FBS) (Invitrogen, USA). The HDF cultures were monitored daily to confirm that there was no contamination.

For characterization experiments, HDF cells from passages two to five were used. The cells were seeded on a 4-well chamber slide (Nunc, Denmark) at a density of 1×10^3^ cell/ml and fixed with cold methanol (Merck, Germany) for 10 minutes at −20°C. Indirect immunofluorescence staining was performed using mouse monoclonal antibodies against human vimentin (Abcam, UK) and human versican (Abcam, UK). The negative control was performed using mouse IgG1 negative control (1:500) (Serotec, USA). Incubations with the primary antibody (1:500) and fluorescent secondary antibody against mouse IgG (1:500) (Abcam, UK) were performed at room temperature for one hour or 45 minutes respectively. To stain the nuclei, 4', 6-diamidino-2-phenylindole (DAPI) (1:500) (Sigma, Germany) was used. Images were captured using a laser scanning microscope (LSM) (Zeiss, Germany).

### Fabrication and characterization of chitosan SRT

Ultrapure medical-grade chitosan powder (Hunza Nutriceuticals, Malaysia) was produced from prawn shell. It was irradiated with 10 kGy of gamma radiation to produce a molecular weight of 440,000 Daltons and dissolved in 0.5 M acetic acid. Distilled water was added and the mixture was heated at 70°C for seven hours in the oven. The mixture was cooled to room temperature, 20% (w/v) glycerol was added and pH was adjusted to 6.2 with 5% (w/v) sodium bicarbonate (NaHCO_3_). The mixture formed a gel to which distilled water was added until the final concentration of the chitosan solution was 2% (w/v). To obtain an inner porous sponge layer, the chitosan solution was poured into a 10 cm by 10 cm polytetrafluoroethylene (PTFE) mold, deep-frozen at −20°C for 24 hours and freeze-dried for 20 hours. The porosity and pore size were controlled by varying the freezing rate which resulted in the formation of ice crystals of varying sizes. Nucleation of the ice crystals was performed by the application of thermal gradients. Removal of the ice crystal by lyophilization generated a porous material. The pore size decreased as the freezing temperature increased. The pores were orientated by controlling the geometry of the thermal gradients during the freezing process.

To determine the pore size, chitosan was punched into small discs that were 15 mm in diameter. The pore diameter was determined at the disc surface by scanning electron microscope (SEM) (Leo, UK). The porosity was calculated using the formula *V*_*m*_*/V*_*p*_ × 100%_,_ where *V*_*m*_ is the volume of the pores and *V*_*p*_ is the volume of chitosan.

To determine biodegradation, chitosan was incubated in PBS containing 1000 unit/ml of lysozyme for 40 days at 37°C. The chitosan was washed with distilled water and freeze-dried. In vitro degradation, *D*, was calculated using the formula *D*= (*W*_*0*_ – *W*_*1*_*)*/*W*_*0*_ × 100%, where *W*_*0*_ is the original weight and *W*_*1*_ is the weight at the time of measurement.

The tensile strength was measured by applying strain at the rate of 0.01 m/min using a universal testing machine (Tinius Olsen, USA). Chitosan of 2 mm thickness was cut into 15 mm × 50 mm sections. The tensile strength, *E* was calculated using the following formula.

where *F* is force, *A* is the cross-sectional area of the chitosan, *ΔL* is its total elongation (change in length) and *L* is its original length.

The water uptake ratio was determined using the formula *W*_*1*_-*W*_*o*_/*W*_*o*_ × 100%, where *W*_*o*_ is the initial weight and *W*_*1*_ is the wet weight after incubating the chitosan in PBS at room temperature for 24 hours.

The water vapor permeability (WVP) of the chitosan was determined using the flexible bottles permeation test (Systech, UK). The bottles were stored at room temperature for 5 hours and the mass of water lost from the bottles was monitored as a function of time. WVP was calculated at steady-state using the formula *WVP* = *W*/*AT* where *W* is the mass of water lost, *A* is the area (1.18 cm^2^) and *T* is the exposure time.

### 3-D cultivation of HDF

For 3-D cultivation, chitosan disc of 5 mm diameter and 2 mm thickness were used. The chitosan discs were put into a 96-well plate and initially seeded with 40 μl of culture medium containing cells at a density of 3×10^6^/cm^2^. The first 20 μl of cells were dispensed using a micropipette onto the center of each chitosan disc. After these cells were adsorbed into the chitosan, the remaining cells were dispensed at its edge. After 2 hours, another 100 μL of culture medium was added to ensure adequate cells proliferation. After 6 hours, the chitosan discs were transferred into 24-well plates to ensure that the cells had enough growth medium for 3-D proliferation. The cultured chitosan discs were incubated in 5% CO_2_ at 37°C. The growth medium was changed every day. On day 14, the chitosan discs with the 3-D cell cultures were harvested for further experiments.

### Attachment of HDF onto chitosan SRT and viability analysis

The viability of cells grown into chitosan was analyzed using a cell viability kit (Invitrogen, USA). The 3-D cell cultures grown into chitosan were incubated with calcein and ethidium, (1:4) in 2 mL PBS to stain live or dead cells respectively. The incubation was performed in 24-well culture plates (Orange Scientific, Belgium) at room temperature for 30 minutes. Chitosan without cells was used as a control. The 3-D cultivated live cells were imaged using a laser scanning confocal microscope (LSCM) (Zeiss, Germany).

To observe the interactions of HDF onto chitosan, the 3-D cells cultures were washed twice with PBS and fixed with 2.5% glutaraldehyde for an hour at 4°C. Serial dehydration using a graded ethanol series of 30, 60 and 100% was performed for 5 minutes each followed by air drying at 37°C for 4 hours. Prior to imaging, the surface of chitosan was sputter-coated with gold (Leica, Czech Republic). Images were captured using SEM (Quanta, Netherlands).

The attachment of cells into 3-D chitosan scaffold was further analyzed by hematoxylin and eosin staining. Briefly, 3-D cultured cells into the chitosan scaffold were fixed with 10% formalin, embedded in paraffin and sectioned at 5 μm thickness. The attachment of cells into the chitosan was viewed using a Mirax Desk scanner (Zeiss, Germany).

To evaluate the viability of cells in 3-D culture, HDF from four independent samples were cultured in a 24-well plate at a density of 3×10^6^/cm^2^. Each well including one with chitosan but without cells as a negative control, was incubated with Presto Blue Cell Viability Reagent (Invitrogen, USA) (1:10) for 24 hours. The medium (100 μl) from each well was transferred into a 96-well plate (Orange, Belgium) and analyzed using a Nano Quant enzyme-linked immunosorbent assay reader (Tecan, Austria). The absorbance was determined at 570 nm with a reference wavelength of 600 nm.

### Statistics

The data are presented as the mean ± SEM.
